# Dominant Optic Atrophy (DOA): Modeling the Kaleidoscopic Roles of OPA1 in Mitochondrial Homeostasis

**DOI:** 10.3389/fneur.2021.681326

**Published:** 2021-06-09

**Authors:** Valentina Del Dotto, Valerio Carelli

**Affiliations:** ^1^Department of Biomedical and Neuromotor Sciences, University of Bologna, Bologna, Italy; ^2^Istituto di Ricovero e Cura a Carattere Scientifico Istituto delle Scienze Neurologiche di Bologna, Programma di Neurogenetica, Bologna, Italy

**Keywords:** OPA1 mutations, dominant optic atrophy, OPA1, mitochondria, cell models, mouse models, iPSCs, retinal ganglion cells

## Abstract

In the year 2000, the discovery of *OPA1* mutations as causative for dominant optic atrophy (DOA) was pivotal to rapidly expand the field of mitochondrial dynamics and describe the complex machinery governing this pathway, with a multitude of other genes and encoded proteins involved in neurodegenerative disorders of the optic nerve. OPA1 turned out to be a much more complex protein than initially envisaged, connecting multiple pathways beyond its strict role in mitochondrial fusion, such as sensing of OXPHOS needs and mitochondrial DNA maintenance. As a consequence, an increasing need to investigate OPA1 functions at multiple levels has imposed the development of multiple tools and models that are here reviewed. Translational mitochondrial medicine, with the ultimate objective of translating basic science necessary to understand pathogenic mechanisms into therapeutic strategies, requires disease modeling at multiple levels: from the simplest, like in yeast, to cell models, including the increasing use of reprogrammed stem cells (iPSCs) from patients, to animal models. In the present review, we thoroughly examine and provide the state of the art of all these approaches.

## Introduction

In the year 2000, the human *OPA1* gene came to attention, as heterozygous mutations were associated with dominant optic atrophy (DOA) (1, 2), a blinding disorder originally described by the Danish ophthalmologist Paul Kjer in 1959, which usually leads to optic atrophy in the first decade of life (3). This gene encodes a protein for which the role as one of the mitochondrial factors involved in the machinery regulating mitochondrial dynamics, promoting fusion of the inner membrane of mitochondria and being key to mitochondrial DNA (mtDNA) maintenance, was clear by analogy with the orthologous genes *MGM1/MSP1* in yeast (4–6). OPA1 was rapidly revealed to have a very complex expression regulation, coming in multiple isoforms due to alternative splicing (7, 8). Only recently have we begun to understand the need for this complexity, revealing how sophisticated and possibly flexible the function of this protein is (9). In fact, besides the role of OPA1 in mitochondrial fusion, by studying OPA1 dysfunction in DOA patients and cell models, its implication in regulating bioenergetics has rapidly emerged (10, 11), contributing to mitochondrial cristae shaping and regulating apoptosis (12, 13), maintaining mtDNA integrity and copy number (14–17), contributing to mitochondrial quality control (18, 19), and ultimately representing a key cross-road for mitochondrial homeostasis, which is central to cell survival and function (20–22).

The identification of *OPA1* mutations as causative for selective neurodegeneration of retinal ganglion cells (RGCs) leading to optic atrophy was thus instrumental in opening a very active field of investigation for neurology and neuro-ophthalmology as well as in understanding new mechanistic pathways regulating mitochondria homeostasis and primarily mitochondrial dynamics (23). Congruently, the expanding spectrum of dominant and recessive mutations affecting the OPA1 protein has been reflected into a progressively larger landscape of clinical phenotypes linked to OPA1 dysfunction, including the vast catalog of the so-called DOA *plus* syndromes (14, 15) dominated by neurodegeneration and multisystem involvement (24), including multiple sclerosis (25, 26), Parkinsonism and dementia (18, 27), infantile Leigh syndrome (28), and cardiomyopathy (17).

Given the constantly growing interest in OPA1 function and dysfunction in relation to human diseases, their modeling is essential to rapidly progress our understanding and possibly provide therapeutic options. Thus, we review the currently available tools for the study of this protein, looking at the future of this rapidly evolving field.

## OPA1 Cell Models

### Yeast Models

**Figure 1:**
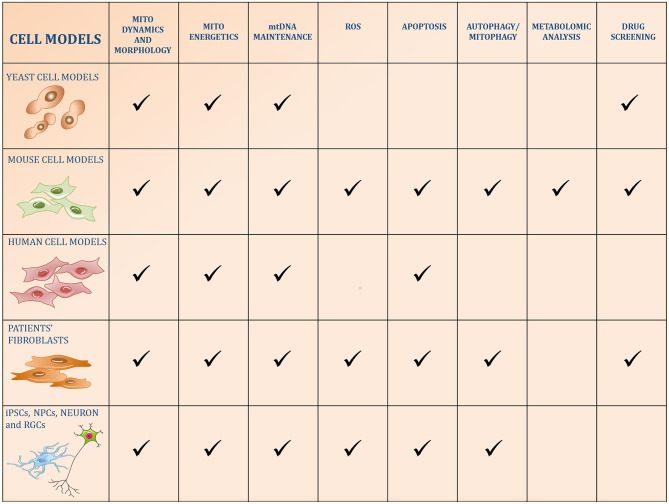
Schematic figure of the five categories of cell models used to study OPA1 functions. The pathways reported in the literature to be regulated by OPA1 or altered by its kd/ko/mutations in the different models are identified by a checkmark.

Yeast, as a simple, easy-to-manipulate eukaryotic organism, is an extremely helpful model with which to understand mitochondrial function, providing insight into mitochondrial pathways and their regulation. Indeed, yeast has been instrumental in identifying key functions of *MGM1* and *MSP1*, the orthologous of *OPA1* in *Saccharomyces cerevisiae* and *Schizosaccharomyces pombe*, respectively (Figure 1). Mgm1 was first identified for its role in mtDNA maintenance (4, 29) and its relative effect on respiratory competence (4, 6). Subsequent reports highlighted the fusion capacity (30–32) and its role for *cristae* structure stability (32, 33). Similarly, Msp1 has been characterized as a protein involved in mtDNA maintenance (5), in mitochondrial network stability and respiratory function (34), and in the fusion of mitochondria (35). Mgm1 is proteolytically processed in two forms, long and short, by the rhomboid-type protease Pcp1, the homolog of the mammalian PARL (36, 37), whereas Msp1 is cleaved by both rhomboid and m-AAA proteases (38). The presence of both short and long forms of Mgm1 is required to maintain a tubular mitochondrial morphology and proper mtDNA amount (36, 39). The increased ratio of long to short form exerts a dominant negative effect on mitochondrial fusion, and a functional GTPase in the short form, but not in the long form, is necessary to tune fusion (39). Furthermore, one of the transmembrane segments of the dynamin Msp1 is required for mtDNA maintenance but not for the fusion activity (40).

Yeast may be used as a model of mitochondrial diseases to investigate the effect of pathogenic mutations on mitochondrial function. Due to the weak conservation of amino acid sequences between Mgm1 and OPA1 and the inefficiency of the human GTPase in complementing *MGM1* depletion, a chimeric protein composed of the N-terminal region of Mgm1 and the catalytic region of OPA1 (41) has been generated. Our group and other investigators used this chimeric model to dissect the effect of several mutations found in DOA patients, showing that pathogenic mutations caused mtDNA loss and mitochondrial fragmentation, reduced the processing of long to short forms, and impaired the respiratory capacity and oxidative growth in yeast (41–43). In addition to the validation of the pathogenicity, the possibility to compare haploid and diploid strains, expressing the mutant only or in combination with the wild-type chimeric protein, allowed for discrimination between hypomorphic, null, or dominant alleles (42).

Yeast is also a valuable system to screen for compounds with therapeutic potential to treat mitochondrial diseases as a “repurposing” strategy. Two research teams applied this approach in the context of *OPA1* mutations (44, 45). In the first screening, a collection of 1,600 repurposed drugs has been used and five compounds were able to suppress the lethality in restrictive conditions caused by a GTPase mutation of *MSP1* in *S. pombe*. Two drugs were able to rescue mtDNA depletion, and one of them recovered also mitochondrial network morphology (44). In the second screening, our group used the yeast strains with mutations on *MGM1* or on the chimeric Mgm1-OPA1 to evaluate the effect of more than 2,500 drugs from two chemical libraries (45). In this case, out of the 26 drugs able to rescue the oxidative growth phenotype, six of them reduced the mitochondrial DNA instability in yeast and were analyzed also in mammalian cell models (45). Therefore, although some differences are present between Mgm1/Msp1 and OPA1, yeast has proven to be a simple and very useful first-line tool for large-scale OPA1 drug screenings, and, coupled with more physiological models to cross-validate the outcomes, it might speed up the discovery of effective therapies for DOA.

### Mouse and Rat Cell Models

The study of OPA1 in mammalian models, such as mouse and rat cells, in particular the mouse embryonic fibroblasts (MEFs), has been instrumental in deepening our understanding of the complex role played by OPA1 in multiple mitochondrial pathways (Figure 1).

Studies in MEFs led to the identification of the OPA1 cleavage sites S1 and S2, encoded by exon 5 and 5b and cleaved by OMA1 and YME1L, respectively, to generate a balanced mixture of long and short forms (46, 47). Recently, a previously suggested third site (48) has been identified, named S3, located in exon 4b, and cleaved by YME1L (49).

Overexpression or silencing of OPA1 in MEFs highlighted its fundamental role in maintaining a filamentous mitochondrial network and sustaining fusion (50, 51), supporting respiratory efficiency (51), and controlling *cristae* integrity and apoptosis (13). However, *Opa1* knockout (ko) mouse fibroblasts have also been extremely helpful in understanding the DOA pathogenic mechanism given the clear-cut phenotype due to the complete lack of the protein. Indeed, *Opa1* null cells have no fusion activity (9, 46, 52, 53), decreased the number of mtDNA and nucleoids (9, 53, 54), and caused profound alterations of *cristae* structure integrity (9, 53, 55). Furthermore, Opa1 ablation and OPA1 overexpression in mouse fibroblasts clarified the interplay between *cristae* and OPA1 in respiratory chain supercomplexe (RCS) assembly (56) and identified the ATP synthase as the effector of the OPA1-mediated protection of mitochondrial functions (57). This model also provided details on the relationship between OPA1 and other interactors to sustain *cristae* architecture, such as the MICOS protein MIC60 (58, 59) and the SLC25A solute carriers family members, which activate the OPA1-mediated *cristae* remodeling in response to different energetic conditions (60).

Moreover, the complete absence of the protein was also instrumental in dissecting two fundamental and debated questions key to OPA1 complexity: the role of the isoforms and of the long and short forms. Expression of each of the eight isoforms in *Opa1* null MEFs disclosed that they are all able to rescue mtDNA content, *cristae* organization, and energetics (9), whereas the short forms are more efficient in restoring energetic efficiency (9, 53) and enhancing cell survival under oxidative stress (61). Importantly, it has been shown that long forms support mitochondrial fusion (9), whereas both long and short forms are necessary to preserve an interconnected mitochondrial network (9, 46, 53). More recently, it has been reported that the presence of multiple isoforms generating different short forms is necessary to achieve a more filamentous mitochondrial network (49), confirming our seminal study (9).

The model of *Opa1* null MEFs has been also employed to study the pathogenic effect of *OPA1* missense mutations associated with DOA in an isogenic genetic background, evaluating the capability to restore mitochondrial defects (42, 62). We substantiated the more severe effect of mutations affecting the GTPase domain when compared to those in the dynamin domain or compared to hypomorphic mutations. This was true for all the mitochondrial readouts analyzed, faithfully mirroring the severity of clinical phenotypes (42). Additionally, these cell models underwent metabolomic and lipidomic studies (63–65). *Opa1* ko MEFs showed bioenergetic changes with altered metabolism of aspartate, glutamate, nucleotides, and NAD (63), and variations in triglycerides and lipids involved in membrane remodeling and in cell signaling pathways (64), similarly to what has been observed in primary *Opa1*-depleted cortical neurons (66). An analysis of OPA1 mutated MEFs ranked the allele severity with the metabolic and lipid alterations, highlighting an increased spermine/spermidine ratio and a reduction in hydroxyproline, amino acid pool, and several phospholipids (65). Interestingly, the increase in glutathione in *Opa1* null MEFs (63) has been recently confirmed in a second study (67) and reported, together with the increased dependency on cysteine transport, as a metabolic adaptive mechanism to afford protection against oxidative stress in these cells (67). Finally, this cell model has been also used for drug screening on amelioration of mitochondrial readouts with different missense mutations, proving to be a valuable tool in testing new DOA therapeutic interventions (45).

### Immortalized and Tumor Human Cell Models

After the discovery that mutations in the *OPA1* gene are the prevailing cause of DOA, several studies have been carried out using immortalized or human tumor-derived cells (Figure 1). These models have been instrumental in elucidating the manifold roles of this GTPase in mitochondrial homeostasis, avoiding the individual variations intrinsic to patient-derived primary cells.

Subcellular localization experiments were instrumental in identifying OPA1 as an inner-membrane protein, exposing the C-terminal portion in the IMS (68–71), which is present as a mixture of long and short forms (71, 72), the latter generated by the activity of the proteases YME1L (72, 73) and OMA1 (48). As the major pathogenic mechanism in OPA1-related DOA is haploinsufficiency, OPA1 downregulation has highlighted mitochondrial network fragmentation (12, 70, 73–75) and complete inhibition of mitochondria fusion (74, 76), confirming its pro-fusion role in mitochondrial dynamics. In addition, several studies found dissipation of the mitochondrial membrane potential (12, 76), drastic disorganization of the *cristae* structure (12, 70, 74, 75, 77), alteration of MICOS assembly (77) and increased sensitivity to apoptosis with the release of cytochrome c (12, 74, 76, 78). The link between OPA1, *cristae* integrity and apoptosis was confirmed in Hek293 cells by expressing a mutant OPA1 resistant to disassembly of its oligomers, which blocked cytochrome c release and apoptosis (79). Downregulation of OPA1 protein also induced mtDNA depletion (16, 75), bioenergetic defect (75, 80), altered Ca^2+^ homeostasis (75, 81) and mitophagy (82). Specific silencing of the three alternative splicing exons has been used in the attempt to unravel a precise mitochondrial function of the individual OPA1 isoforms (16, 83).

The pro-fusion role of long forms has been reported in several studies, where overexpression of long OPA1 was more efficient compared with the long/short combination in ameliorating the mitochondrial network morphology in knockdown (Kd)-*OPA1* cells (71) and in SH-SY5Y cells after hypoxia and re-oxygenation injury (84). Furthermore, it has been reported that SIRT4 overexpression increased OPA1 long-form promoting fusion, counteracting fission and mitophagy (85).

Human cells have been used also to confirm the new function identified in adipose-like mouse fibroblasts (86), where OPA1 localized in lipid droplets serves as an A-kinase anchoring protein (AKAP) and allows PKA to phosphorylate perilipin 1 to favor lipolytic stimulation (87).

### Patients' Derived Fibroblasts and Lymphoblasts

Fibroblasts and lymphoblasts derived from patients are a model extensively used to study the pathophysiology of OPA1 mutations. The majority of the alterations identified in the previous models have been reconfirmed in patients' cells (Figure 1). Numerous studies of fibroblasts and lymphoblasts reported defective mitochondrial network dynamics (11, 18, 27, 42, 78, 88–95), energetic metabolism (11, 42, 88, 91, 93, 94, 96), *cristae* structure maintenance (11, 27, 93), and increased sensitivity to apoptosis stimuli (11, 96). Depletion of mtDNA copy number in fibroblasts has been reported in a few cases in the presence of missense (42, 96) or compound heterozygous mutations (19, 43).

Recently, increased ROS production (94, 96), low levels of antioxidant enzymes (97), and alteration of calcium uptake (98) have been also reported. Furthermore, direct involvement of OPA1 in quality control has been revealed in fibroblasts (18, 19, 95), where missense mutations induced an increase in both autophagy and mitophagy processes. Interestingly, basal mitophagy was increased in fibroblasts bearing dominant-negative *OPA1* mutations, whereas OPA1 haploinsufficiency seems to correlate with a reduction in mitochondrial turnover and autophagy (95).

Although these cells have been very useful when studying mitochondrial alterations, the presence of the wild-type allele often hides the effect of the mutated one, prompting the need to use galactose media to force oxidative metabolism and reveal the mitochondrial dysfunctions. Also, nuclear and mitochondrial genomes may variably contribute to the great heterogeneity observed in clinical and cellular phenotypes due to the same *OPA1* mutation. For example, analysis of mtDNA copy number revealed a depletion only in one of two fibroblasts with the same R445H mutations derived by patients belonging to the same family (42), suggesting that other genetic or environmental factors may contribute. Concordantly, mitochondrial OMI/HTRA2 has been recently reported as a new gene modifier of phenotype variability (99).

Although with these limitations, fibroblasts, if combined with other models, may be functional in identifying new pathways, confirming pathogenic mechanisms, or validating the efficacy of therapeutic molecules, as we recently reported (45).

### iPSCs, NPCs, Neuron, and RGCs

In the last decade, the establishment of induced pluripotent stem cell (iPSC) technology has provided the opportunity to generate *in vitro* human models of neurological disorders. The iPSCs are stem cells-like reprogrammed *in vitro* from patient-derived primary cells, which can be then differentiated into specific somatic cell types (100). The iPSCs and terminally differentiated cells, therefore, allow us to study the exact cell type affected in the human disease to identify the pathological mechanism and to conduct drug screening on a human genetic background (Figure 1).

Human iPSCs lines have been generated from patients' fibroblasts carrying different *OPA1* mutations, such as the p.Gln621Ter (101) and the p.Ser545Arg (102) heterozygous mutations, and the compound heterozygous mutations causing Behr syndrome (103). All these iPSCs could differentiate into the three germ layers (endoderm, mesoderm, and ectoderm) (101–103).

Increased apoptosis and inefficient capability to differentiate into a neural rosette and RGCs were reported for the iPSCs derived from two other fibroblasts of patients carrying the intronic mutation c.2496+1G>T, suggesting the impact of apoptosis on RGCs possibly leading to early or congenital optic atrophy (104), as previously proposed by optical coherence tomography clinical studies (105). The addition of the neural induction medium, the secreted signaling molecule noggin, or the estrogen hormone promoted the differentiation into RGCs of the *OPA1*^+/−^ iPSCs, possibly by inhibiting apoptosis (104).

Dopaminergic neurons carrying an *OPA1* mutation causing haploinsufficiency were generated via iPSCs from two patients belonging to the same family that, interestingly, developed different clinical phenotypes: isolated DOA or DOA with syndromic Parkinsonism (106). Both the cell lines showed a reduction in oxygen consumption rate (OCR), complex I levels, and activity, whereas only neurons derived from the patient with Parkinsonism presented mitochondrial fragmentation and an increase of the OPA1 short forms. In this study possible genetic modifiers of the different clinical phenotypes in the two patients, presenting the same OPA1 mutation, were not identified (106).

Neural progenitor cells (NPCs) from two *OPA1* missense mutations causing Parkinsonism and dementia (18) presented bioenergetic defect, mitochondrial network fragmentation, increased ROS levels, and alteration in the lysosome pathway (107). Even in this case, the *OPA1*-mutated NPCs showed a survival deficit, which was rescued by selective inhibition of necroptosis (107). The iPSC-derived neurons bearing these two *OPA1* mutations have been further studied in a microfluidic system where different neuronal subtypes were cultured together with a patterned organization of their projections and synaptic terminals (108). Analysis of neuronal projections revealed altered content, distribution, and movement of mitochondria along the axons of the neurons. Moreover, the impairment in synapse formation was followed by a progressive loss of synaptic contacts over time in the mutant neurons, suggesting a depletion of mitochondria in neuronal projections as a cause of loss of neuronal connectivity and neurodegeneration (108).

Recently, human embryonic stem cells (hESCs) with OPA1 haploinsufficiency induced by the CRISPR-Cas9 technology have been characterized and differentiated into NPCs. Interestingly, several genes involved in NPCs differentiation, GABAergic interneuron formation, and retinal development were downregulated in *OPA1*^+/−^ NPCs due to increased CpG methylation (109). The increased ROS levels and downregulated FOXG1 expression, a factor crucial for GABAergic neuronal formation and retinal development, were confirmed also in NPCs derived from two other patients bearing the common c.2873_2876delTTAG mutation, suggesting that OPA1 haploinsufficiency may result in aberrant nuclear DNA methylation and an altered transcriptional program (109).

Although studying the cells targeted by the disease accelerates our understanding of OPA1-derived dysfunctions, these two-dimensional methods to studying neuronal dysfunction do not completely recapitulate the complexity of a human brain (110). The recently optimized protocols for modeling *in vitro* brain and retina organogenesis (111) represent a further fundamental step instrumental in disease modeling.

## Opa1 Animal Models

### *Drosophila melanogaster* Models

**Figure 2:**
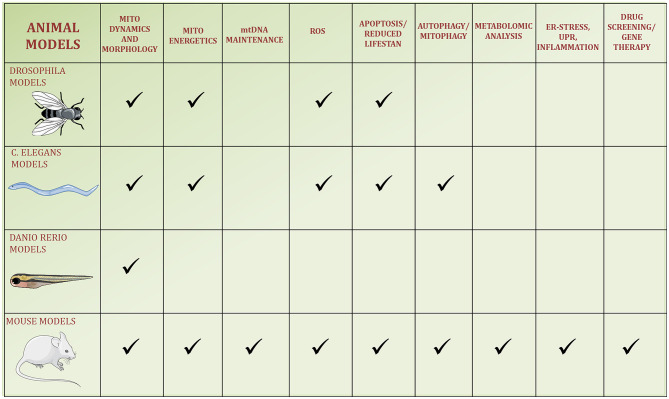
Schematic figure of the four animal models used to study OPA1. The pathways reported in the literature to be regulated by OPA1 or altered by its kd/ko/mutations in the animal models are identified by a checkmark.

Modeling of DOA has also been carried out in *Drosophila melanogaster* (Figure 2) and these studies emphasized the role of increased ROS production in leading to the pathologic phenotype, showing how this could be partially counterbalanced by antioxidant therapy (112). This supports the proposal of therapeutic approaches with antioxidants in human patients with DOA. The heterozygous mutant flies showed an age-dependent perturbation of visual functions, heart alterations (113), lifespan reduction, elevated ROS level, and the presence of irregular and dysmorphic mitochondria in the skeletal muscle (114). In summary, these two studies on the *Opa1* mutant *Drosophila* model showed an age-dependent multisystemic disorder, resembling the syndromic forms of DOA “plus” (113, 114).

### *Caenorhabditis elegans* Models

The tight interconnection between OPA1 mutations and ROS alteration has been further confirmed in *Caenorhabditis elegans* (*C. elegans*) models. Indeed, the mutations in the *eat-3* gene, the *OPA1* worm orthologous gene, increased susceptibility to damage from free radicals, as shown by increased sensitivity to paraquat and to the loss of the mitochondrial superoxide dismutase sod-2 (115). Transgenic worms carrying loss of function *eat-3* mutations presented fragmented mitochondria (115–117) and aberrant *cristae* architecture (115, 117). The *eat-3* mutation caused an age-dependent and progressive deficit in movement, as well as in muscle and neuronal function (117), somehow mirroring the DOA “plus” clinical phenotypes. Interestingly, increased levels of elongated mitochondria have been linked to longevity, as mitochondrial fusion allows the survival of older animals (118), a finding supported by a second study showing that *eat-3* mutation reduced median animal lifespan (117), as already reported in the *Drosophila* model (114).

The increased autophagy/mitophagy as a key pathogenic mechanism in DOA has been confirmed also in *C. elegans*, where the expression of mutated OPA1 in GABAergic axons reduced mitochondrial content in axons, a phenotype that was counteracted by depletion of the *ATG8* homolog *lgg-2* (119).

### *Danio rerio* (Zebrafish) Models

The effect of Opa1 depletion in the early development has been studied in a *Danio rerio* model using the antisense morpholino (120). The Opa1 morphants showed developmental delay, decreased blood circulation velocity, reduction of the eye size and heart rate, defects associated with increased mitochondrial fragmentation in muscle cells, and impaired bioenergetics (120).

The alteration of mitochondrial networks in Opa1 morphants has been reported more recently (121), confirming the main role of OPA1 in mitochondrial morphology.

### Mouse Models

Three mouse models carrying a heterozygous germline mutant *Opa1* allele have been reported to date and have been instrumental in studying the pathogenic mechanisms of DOA (Figure 2) (122–124). Screening an ENU-mutagenized DNA library of mouse DNA led to generating two of the murine models, both recapitulating the human genetic defects that induce haploinsufficiency. The first, B6;C3-*Opa1*^329−355*del*^ mutant mouse, leads to a splice error with the skipping of exon10, which ultimately causes an in-frame deletion of 27 amino acid residues in the dynamin GTPase domain (122). The second, B6;C3-*Opa1*^*Q*285*STOP*^ mutant mouse, results in a truncated protein (123). Both models have about a 50% reduction in OPA1 expression and the homozygous condition is embryonically lethal, pointing to the crucial role played by OPA1 during fetal development. The third model is a knock-in mouse carrying the common *OPA1 c.2708_2711delTTAG* mutation in humans in a C57Bl6/J mouse background (124). In these mice, a 25% reduction in OPA1 protein was recognized in the brain, retina, optic nerve, and glycolytic fibers, whereas the reduction reached 50% in oxidative fibers and heart. Also, in this model, the homozygous condition was embryonically lethal.

In all three models, the heterozygous animals displayed a mild, age-dependent ocular phenotype with well-documented RGCs dysfunction and loss (122, 124–126). At histopathology, degenerative features were observed in the optic nerves including demyelination, various degrees of axonal degeneration, and abnormalities of mitochondria at the ultrastructure level (123–126).

Increased autophagy was reported in the RGCs of the B6;C3-*Opa1*^*Q*285*STOP*^ mutant mouse (127) and in the glycolytic fibers, RGCs, and peripheral neurons of the *Opa1*^*delTTAG*^ mutant mouse (124), highlighting the autophagic elimination of mitochondria with impaired fusion. More recently, increased mitophagy has been reported in the B6;C3-*Opa1*^*Q*285*STOP*^ mice (128) and confirmed in mouse RGCs overexpressing a mutated OPA1, showing also that reduced axonal mitochondrial density was linked to increased autophagic mitochondrial degradation in the RGCs soma, in close proximity to axonal hillocks (119). The B6;C3-*Opa1*^329−355*del*^ mice were also reported to present an imbalance of redox state possibly increasing mitochondrial ROS, as suggested by the decrease in aconitase activity and induction of antioxidant defenses (97). Metabolic analysis of the optic nerve in female *Opa1*^*delTTAG*^ mice identified changes in the concentrations of metabolites involved in neuroprotection and of phospholipids, some of them suggestive of myelin sheath alteration (129). All these dysfunctions may lead most RGCs to death but not the melanopsin-expressing RGCs, which are reported to survive in two DOA mice models (130, 131) according to evidence that also in humans this cell type is relatively resistant to cell death in mitochondrial optic neuropathies (132).

In summary, the phenotype in these mouse models resembles sufficiently the human disease, characterized by loss of RGCs and optic nerve atrophy. In humans, the disease may vary in clinical severity, from severe congenital cases to very mild, subclinical disease disclosed only by accurate ophthalmological investigations (133, 134). Interestingly, systemic examination of these animals revealed mild neuromuscular impairment, including decreased locomotor activity, abnormal clutching reflex, and tremor, in analogy to the continuum clinical spectrum in humans, ranging from DOA to DOA “plus” (134).

Remarkably, interesting results were obtained by a closer investigation of the RGCs synaptic connectivity in the B6;C3-*Opa1*^*Q*285*STOP*^ mutant animals, looking at their dendrites instead of focusing only on axons (135, 136). Counterintuitively, the earliest pathological changes occurred in RGCs dendrites, showing pruning and marked reduction in their synaptic connectivity (135, 136). This dendropathy is, however, congruent with the crucial role played by OPA1 and mitochondrial fusion in maintaining dendrites and their synapses (137).

Furthermore, with aging impaired cardiac function has been reported in all three mice models. Indeed, at onset of blindness, the aged B6;C3-*OPA1*^*Q*285*STOP*^ mice also showed cardiomyopathy, characterized by disruption of mitochondrial organization, mtDNA depletion, bioenergetic defect, and defective cardiac mitochondria (138). Reduction in cardiac adaptation to pressure overload and heart hypertrophy were observed also in the B6;C3-*Opa1*^329−355*del*^ mice (139). Similar alterations in mitochondrial calcium handling affected these *Opa1* mutant mice (139), observed also in the third *Opa1*^*delTTAG*^ mice, together with increased sensitivity to cardiac ischemia/reperfusion injuries (140). Interestingly, cardiac involvement in patients carrying *OPA1* mutation has been reported for the first time in two patients harboring a homozygous recessive *OPA1* mutation leading to a fatal encephalopathy with progressive hypertrophic cardiomyopathy (17).

A further mouse model with deleted *Opa1* in pancreatic beta cells showed glucose intolerance and impaired insulin secretion. It also presented a reduced glucose-stimulated ATP production and developed hyperglycemia. Focusing on mitochondrial function, the beta cells presented a severe alteration of mitochondrial structure and a reduction of subunits' level and activity of Complex IV (141).

Recently, muscle-specific *Opa1* ablation mice models have been generated to investigate the tissue-specific role of *Opa1* in muscle, highlighting its metabolic role (142–144). Pereira et al. showed that in mutant mice the progressive mitochondrial dysfunction led to an increased metabolic rate, muscle atrophy, and insulin resistance induced by a mechanism involving ER stress and secretion of fibroblast growth factor 21 (FGF21) (142). Increased FGF21 has been observed also in another muscle-specific *Opa1*-deletion mouse model, where, together with ER stress and activation of the unfolded protein response (UPR), a catabolic program with muscle loss was activated, leading to systemic aging and premature death (143). In a third study, the enhanced FGF21 level and premature death elicited by *Opa1* ablation were associated with muscle inflammation characterized by NF-kB activation and increased expression of pro-inflammatory genes; these features were blocked by mtDNA depletion and repression of TLR9 (144). All these features are of great interest, as plasma cytokines, including FGF21, have been recently validated as biomarkers for mitochondrial diseases, especially those with prominent muscle involvement (145, 146).

From a therapeutic point of view, all these mice models are instrumental in testing drugs or therapies. Indeed, the *Opa1*^*delTTAG*^ mouse has been already tested for OPA1 isoform 1 gene therapy, proving to mitigate the OPA1-induced RGCs degeneration, encouraging a possible clinical translation in DOA patients (147). Furthermore, the most recent mouse model with RGC-specific overexpression of mutant OPA1 was instrumental in showing that contrasting the excess autophagy by various strategies was effective to avoid RGCs degeneration and restore vision (119).

## *In vitro* Models

Over the last decade, to elucidate the OPA1 role in the fusion of the inner mitochondrial membrane, various *in vitro* assays with purified OPA1 have been developed. Initially, the simpler yeast mgm1 has been studied by purifying short Mgm1 (s-Mgm1), revealing that the protein exhibits GTP activity, self-assembles into low order oligomer, and interacts specifically with negatively charged phospholipids present in the mitochondrial membranes (148). Furthermore, it has been reported that s-Mgm1 oligomerization and its binding to mitochondrial phospholipids strongly stimulates its GTPase activity, and it assembles onto liposomes and boosts liposome interaction, indicating that s-Mgm1 can tether opposing membranes mediating their fusion (149). This hypothesis was supported by cryo-electron microscopy studies and liposome fusion assays, showing that s-Mgm1 self-associates to tether opposing membranes with a gap of 15 nm and the oligomers undergo GTP-dependent structural changes that may induce fusion (150). Together with the ability to cause phospholipid clustering, s-Mgm1 was also reported to trigger local membrane bending and the formation of tubular structures (151). The investigations of both long and short Mgm1 forms in fusion highlighted that both forms are preferentially inserted into liposomes containing the lipid cardiolipin acting together in trans to form a functional unit required for mitochondrial fusion (152).

Similar results have been obtained by studying the molecular mechanisms of OPA1-mediated fusion. Indeed, the association with liposomes containing negative phospholipids, such as cardiolipin, enhanced the GTPase activity of short forms of OPA1, promoted their assembly into oligomers, and led to membrane tubulation, activities that were selectively impaired by DOA mutations (62). Using an *in vitro* purified mitochondria system, it has been shown that mitochondrial fusion is dependent on proteolytic processing of long forms (153). To disentangle the debated issue of the fusogenic capability of OPA1 forms, Ban et al. purified and analyzed short and long forms (154). The *in vitro* membrane fusion assays revealed that long forms and cardiolipin on opposite sides cooperate in mitochondrial inner membrane fusion, a process that is efficiently accelerated by a defined amount of short form and lowered by its excess level. On the other hand, a cardiolipin-independent interaction of long forms located on opposite membranes was reported to promote membrane tethering, thus sustaining the *cristae* structure (154). Concordantly, an *in vitro* reconstitution system, able to distinguish the sequential steps in membrane fusion, disclosed that the short form mediates membrane tethering, the long form is sufficient for membrane docking, hemifusion and low levels of content release, and the short form cooperates with the long form to mediate efficient and fast membrane pore opening (155). Importantly, as seen in other studies, the excess levels of short form inhibited fusion activity (155). Cryo-electron microscopic structures revealed that the short form presents the classic dynamin-like structure, can bind to membranes, induces membrane tubulation by forming a helical array, and GTPgS binding promotes changes in S-OPA1 assembly from a “closed” to an “open” conformation (156).

New mechanistic insight into how OPA1/Mgm1 mediates the membrane fusion has been provided by the recently solved crystal structures of short Mgm1 from *Chaetomium thermophilum* (157) and *Saccharomyces cerevisiae* (158). The fungus short Mgm1 was reported to form stalk-mediated tetramers and assemble on positively or negatively curved membranes (157), whereas the yeast short Mgm1 forms a concave membrane-associated head-to-tail trimeric structure built by intermolecular interactions (158). The crystal structure of the minimal GTPase domain (MGD) of OPA1 and biochemical analysis, instead, revealed that it can form nucleotide-dependent and -independent dimers, which may combine to form higher-order oligomers (159).

All these *in vitro* findings resolve some conflicting results obtained with cellular studies and helped to highlight the complexity of OPA1 activities, fundamental to maintain and support the inner membrane structure and fusion.

## Conclusions

The investigation of OPA1 function has witnessed a tremendous deal of progress in the last decade thanks to a multitude of new models and approaches that we here reviewed. This progress was instrumental in refining the role of mitochondria in RGCs survival and to deepen our understanding of DOA pathogenesis to possibly establish effective therapies for these patients, currently an unmet need that urges a research effort. A few *proof of principle* studies already provided interesting results, such as by pharmacologically or genetically limiting the overactive autophagy in RGCs (119), or refining strategies based on gene therapy approaches (147). The multifaceted role of OPA1 in mitochondrial function and, more generally, in cell metabolism continues to surprise, and the possible therapeutic role of slight OPA1 overexpression has also been explored to counteract an array of different conditions (160–162), which is well-beyond the specific field of inherited optic neuropathies.

## Author Contributions

VD and VC contributed to the writing of the manuscript. Both authors contributed to the article and approved the submitted version.

## Conflict of Interest

The authors declare that the research was conducted in the absence of any commercial or financial relationships that could be construed as a potential conflict of interest.
